# Effect of prolyl hydroxylase domain 2 haplodeficiency on liver progenitor cell characteristics in early mouse hepatocarcinogenesis 

**DOI:** 10.17179/excli2016-607

**Published:** 2016-11-11

**Authors:** Eliene Bogaerts, Annelies Paridaens, Xavier Verhelst, Peter Carmeliet, Anja Geerts, Hans Van Vlierberghe, Lindsey Devisscher

**Affiliations:** 1 Department of Gastro-Enterology, Ghent University, Ghent, Belgium; 2 VIB Vesalius Research Centre, KU Leuven, Leuven, Belgium

**Keywords:** hepatocarcinogenesis, hypoxia, liver progenitor cells, notch, prolyl hydroxylase domain, diethylnitrosamine

## Abstract

Activation of the hypoxia-inducible factor (HIF)-pathway in hepatocellular carcinoma (HCC) induces therapy resistant tumours, characterized by increased liver progenitor cell (LPCs) characteristics and poor prognosis. We previously reported corresponding results in mice with HCC in which hypoxia was mimicked by prolyl hydroxylase domain (PHD) inhibition. Here, we aimed at investigating whether induction of LPC characteristics occurs during the onset of hepatocarcinogenesis and if this is associated with activation of Notch signalling. Dietheylnitrosamine (DEN) was used to induce hepatic tumours in PHD2 haplodeficient (PHD2^+/-^) mice which were euthanized at 5, 10, 15 and 17 weeks following DEN during neoplastic transformation, before tumour formation. Neoplasia and mRNA expression of LPC and Notch markers were evaluated by histology and qPCR on isolated livers. PHD2 haplodeficiency resulted in enhanced expression of HIF target genes after 17 weeks of DEN compared to wild type (WT) littermates but had no effect on the onset of neoplastic transformation. The mRNA expression of Afp and Epcam was increased at all time points following DEN whereas CK19, Prom1 and Notch3 were increased after 17 weeks of DEN, without difference between PHD2^+/-^ and WT mice. MDR1 mRNA expression was increased in all DEN treated mice compared to saline control with increased expression in PHD2^+/- ^compared to WT from 15 weeks. These results indicate that the effects of PHD2 haplodeficiency on the expression of LPC and Notch markers manifest during tumour nodule formation and not early on during neoplastic transformation.

## Introduction

With an estimated overall five year survival of less than 20 %, liver cancer is the 2^nd^ leading cause of cancer related death worldwide (Jemal et al., 2011[19]). Liver tumours often arise in a background of chronic liver disease characterised by inflammation, sinusoidal capillarisation and the formation of fibrous septa. Moreover, when tumours outgrow their vascular supply, newly formed vasculature is often structurally and functionally anomalous during further tumour growth. These processes contribute to a reduced liver oxygenation early during tumour development and later, during tumour growth (Yang and Poon, 2008[38]; Heindryckx et al., 2012[16]). 

Insufficient oxygen supply results in hypoxia, a situation known to inhibit prolyl hydroxylase domain (PHD) enzyme activity, causing stabilisation of the hypoxia inducible factor (HIF). HIF stabilisation and its nuclear translocation results in the transactivation of genes involved in cell-survival by, amongst others, stimulating (neo-)angiogenesis (through induction of pro-angiogenic factors, such as the vascular endothelial factor or Vegf), and boosting the anaerobe glucose metabolism (via Glucose transporter 1 or Glut1, phospho-fructokinase or Pfk) (Heindryckx et al., 2012[16]; Bogaerts et al., 2014[2], 2015[1]). Activation of the hypoxia inducible pathway is known as the 'hypoxic adaptive response' and has extensively been investigated in tumorigenesis as a mediator of tumour growth, therapy resistance and metastasis (Harris, 2002[15]; Lu and Kang, 2010[25]; Lin and Wu, 2015[24]) 

Previous studies have shown that activation of the hypoxic pathway can induce therapy resistance and is related to poor prognosis in primary liver tumours (Comerford et al., 2002[6]; Paez-Ribes et al., 2009[30]; Lu et al., 2010[25]; Liang et al., 2013[23]; Luo et al., 2014[26]; Yang et al., 2014[37]). Furthermore, in humans, pre-operative trans-arterial chemoembolization, which has been shown to induce a hypoxic adaptive response, has been linked to higher recurrence rates and a phenotypic switch from HCC to HCC-CC, with increased expression of liver progenitor cell (LPC) characteristics (Zen et al., 2011[39]; Zeng et al., 2012[41]; Fang et al., 2013[10]). In accordance, we previously reported that inhibiting PHDs, (using a pan-PHD inhibitor or PHD2 haplodeficient mice (PHD_2_^+/-^)), in murine diethylnitrosamine (DEN)-induced hepatocellular carcinoma (HCC), results in a more aggressive mixed hepato-cholangiocarcinoma (HCC-CC) phenotype high in liver progenitor cell (LPC) characteristics, coinciding with increased expression of markers for metastasis and actors of the Notch signalling pathway (Heindryckx et al., 2012[16]; Bogaerts et al., 2015[1]). Possibly, PHD inhibition during carcinogenesis can readily prime future tumour cells to react differently to later hypoxic stimuli, and give rise to more aggressive mixed phenotype cancers, with increased LPC characteristics and an increased risk for therapy resistance and metastasis (Bogaerts et al., 2014[2]). 

LPCs are bipotential cells that reside in the canals of Hering in the liver, were they act as facultative adult stem cells (Spee et al., 2010[34]). In healthy liver, loss of hepatocyte or cholangiocyte cell mass can easily be replaced by the immense self-replicative capacity of the parenchyma. However, in situations of severely reduced liver function, like in chronic liver disease, the progenitor cell compartment is activated (Boulter et al., 2012[3]). 

LPCs then proliferate and migrate to the site of injury where they differentiate to replenish the lost cell mass by a series of tightly organised interactions controlled by the Notch and Wnt signalling pathways. Activation of the Notch pathway drives LPC's towards a cholangiocytic phenotype, while Wnt induced inhibition of Notch signalisation results in hepatic differentiation (Boulter et al., 2012[3]).

The Notch pathway not only plays a pivotal role in the cell- fate determination of LPCs, it is also shown to be an important mediator of hepatocarcinogenesis. Interestingly, activation or inhibition of the different Notch receptors can have both pro- and anti- oncogenic effects (Dill et al., 2013[8]; Ortica et al., 2014[29]; Geisler and Strazzabosco, 2015[11]; Huntzicker et al., 2015[18]). In our previous studies, mRNA expression of actors of the Notch signalling pathway was increased in DEN induced HCC in which PHDs were inhibited. The Notch pathway could thus play a role in PHD inhibition-mediated expression of LPC characteristics, which would be an attractive therapeutic target. 

Since we observed increased expression of LPC characteristics by inhibiting PHD proteins during HCC development, which was associated with increased mRNA expression of actors of the Notch pathway, we aimed to investigate if the effect of PHD2 haplodeficiency on liver tumour phenotype in advanced DEN induced HCC is preceded by altered LPC and/or Notch expression at early stages of hepatocarcinogenesis. A better understanding of the effect of hypoxic conditions early during tumour initiation and development, mimicked by PHD2 haplodeficiency, a situation readily present during chronic liver disease and tumour relapse, could allow us to pinpoint critical markers and events involved in the observed hypoxia induced phenotypic switch, therapy resistance and metastasis.

## Materials and Methods

### Induction of hepatocarcinogenesis in PHD_2_ haplodeficient mice

PHD_2_^+/-^ mice were obtained from the Vesalius Research Center (KUleuven, Leuven, Belgium). A heterozygous couple was used for breeding and offspring was genotyped using the following primers in a concentration of 10 µM: ACCTATGATCTCAGCATTTGGGAG, TCAGGACAGTGAAGCCTAGAAACT and AAATTCTAATCGTAGCTGATGTGAGC (Heindryckx et al., 2012[16]).

To investigate the effect of PHD2 haplodeficiency on early hepatocarcinogenesis in mice, 5 week old PHD_2_^+/- ^and wild type (WT) littermates (129S6 background) received weekly intraperitoneal DEN injections (35 mg/kg, Sigma-Aldrich, Bornem, Belgium). This induces microscopic neoplastic cells after 15 weeks, macroscopic nodule formation at 20 weeks and HCC after 25 weeks, which was previously reported by our group (Heindryckx et al., 2010[17]). These mice were euthanized after the 5^th^, 10^th^, 15^th^ and 17^th^ week of DEN, before HCC nodules could form (Heindryckx et al., 2010[17]). As we have previously shown that there is no difference between WT and PHD_2_^+/-^ healthy mice (Heindryckx et al., 2012[16]), we administered weekly saline injections for 17 weeks to PHD_2_^+/- ^mice as controls.

Mice were euthanized at indicated time-points by cervical dislocation, the liver was excised and divided for histology and qPCR analysis, respectively submerged in 4 % formaldehyde (Klinipath, Olen, Belgium) for paraffin embedding and stored at -80 °C in RNA later (Ambion, Thermo Fisher scientific, Gent Belgium) for RNA extraction.

All experiments were approved by the ethical committee for animal experiments at the faculty of medicine and health sciences of Ghent University Belgium (ECD13/61).

### Histological evaluation

General morphology of liver tissue was assessed using Haematoxylin-Eosin, Sirius Red and Reticulin stainings on 5 µm sections of paraffin embedded tissue as routinely described. Neoplasticity was defined as enlarged cells with normal nucleus to cytoplasm ratio (n/c), small cells with increased n/c, enlarged pleomorphic nuclei, and binucleation, (pre) neoplastic hepatocytic lesions were identified by loss of reticulin staining and sirius red staining was performed to identify potential cholangiocytic lesions marked by cholangiofibrosis, as previously described (Bogaerts et al., 2015[1]). 

Cytokeratin 19 immunohistochemistry (1/200 in TBS, ab133496, RRID: AB_11155282, abcam, Cambridge, UK) was used to visualize structures of the cholangiocytic lineage as well as LPCs. Overall CK19 immunoreactivity was measured using Cell D software (Olympus Imaging Solutions, Münster, Germany) and to evaluate the LPC response, 5 portal areas were centred at a magnification of 400 and all CK19 positive single cells were counted. 

### Quantitative real time PCR (qPCR)

RNA was extracted from 20 mg of frozen liver tissue preserved in RNA-later, according to the manufacturer's guidelines (Rneasy Mini Kit, Quiagen, Venlo, the Nederlands).

cDNA was obtained from 1 µg RNA using the iScript cDNA synthesis kit (Bio-Rad, Nazareth-Eke, Belgium) and quantitative PCR (qPCR) analyses were performed using the Lightcycler 480 Green I master mix (Roche, Vilvoorde, Belgium), using the primersets listed in Table 1(Tab. 1).

All reactions were run in duplicate; the comparative Cq method was used to determine the number of transcripts which were normalised to reference genes that showed stable expression in all samples, as also previously described (Heindryckx et al., 2012[16]; Bogaerts et al., 2015[1]). 

### Statistical analysis

Data were analysed using SPSS23 software (IMB corp, Armonk NY, USA) and graphs were illustrated using Graphpad prism 6 software (Graphpad software, inc; San Diego CA, USA). Kolmogorov-Smirnov test was used to test for normality. Student's T- test was then performed in case of normality; the Mann-Whitney-U test was used for not normally distributed data. P-values ≤ 0.05 were considered significant. All data are presented as mean ±SEM.

## Results

### PHD_2_ haplodeficiency does not alter the onset of neoplastic transformation 

**Figure 1:**
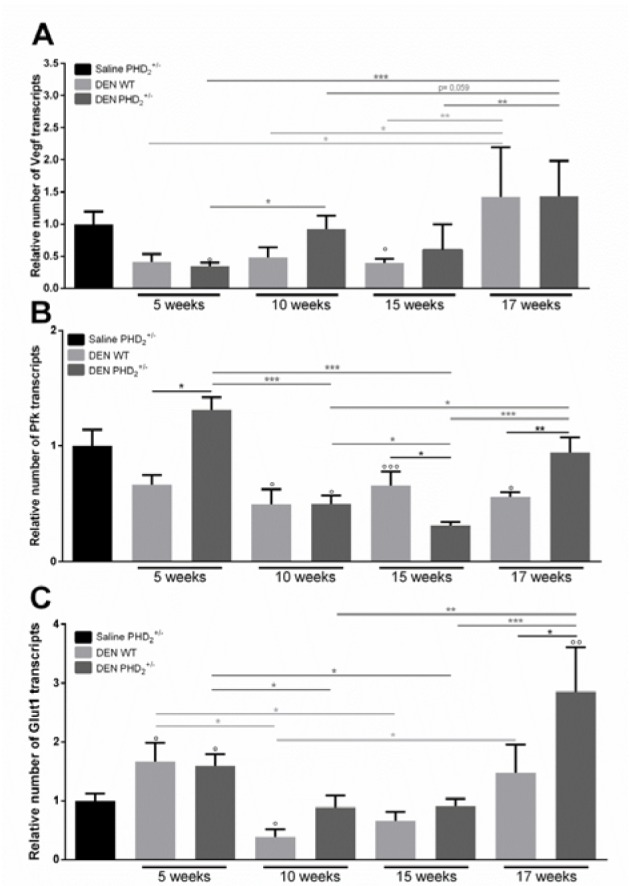
mRNA expression of HIF target genes A. vascular endothelial growth factor alpha (Vegfa), B. phosphofructokinase (Pfk) and C. glucose transporter 1 (Glut1) in PHD_2_^+/-^ and WT mice, euthanised at different time points in hepatocarcinogenesis. ° p < 0.05, °° p < 0.01 and °°° p < 0.001 compared to saline control mice * p < 0.05, ** p < 0.01 and *** p < 0.001

We first assessed the effect of PHD_2_ haplodeficiency on HIF stabilisation by assessing the activation of HIF target genes. In early hepatocarcinogenesis, we did not observe a significant activation of the hypoxic pathway compared to saline control mice, in either genotype. However, HIF downstream targets showed a peak RNA expression after 17 weeks of DEN compared to other time points (Figure 1A, C(Fig. 1)) and in PHD_2_^+/-^ mice compared to WT mice at the same time-point. (Figure 1B, C(Fig. 1)). 

To assess general morphology and neoplasia, haematoxylin-eosin, sirius red and reticulin stainings were performed. Neoplastic cells and reticulin free hepatocytic plates could be observed from 10 weeks onwards (Figure S1) and neoplastic nodules were observed from 15 weeks onwards (Figure S1). Sirius red staining was evaluated as previously described (Bogaerts et al., 2015[1]), and showed no cholangiocytic lesions (Figure S1). No difference was observed between PHD_2_^+/-^ and WT livers at the indicated time points.

### Neoplastic transformation coincides with increased expression of LPC characteristics during early hepatocarcinogenesis 

To evaluate the effect of PHD2 haplodeficiency on the expression of LPC characteristics, we performed qPCR analysis of Cytokeratin 19 (CK19), Prominin 1 (Prom1), Epithelial cell adhesion molecule (Epcam), Alpha fetoprotein (Afp) and multi drug resistance protein 1 (MDR1). 

**Figure 2:**
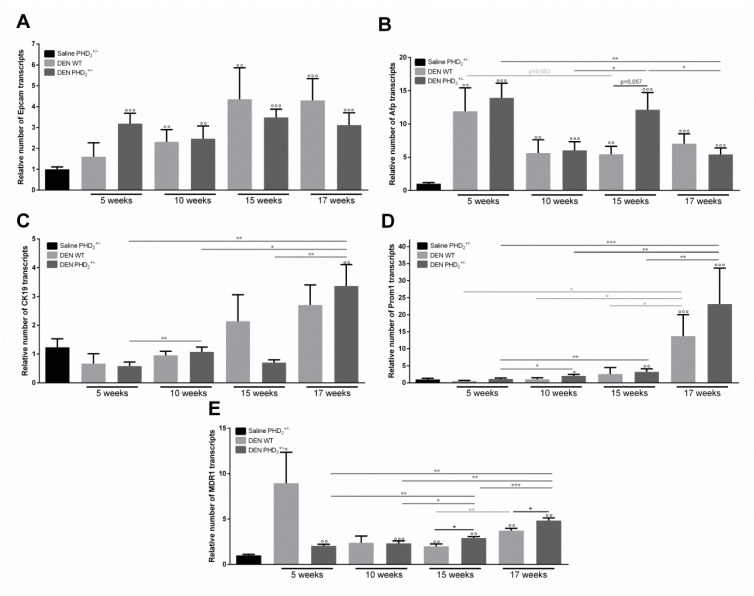
mRNA expression of LPC markers A. epithelial cell adhesion molecule (Epcam), B. alpha feto-protein (Afp), C. cytokeratin 19 (CK19), D. prominin 1 (Prom1) and E. multi drug resistance protein 1 (MDR1) in PHD2+/- and WT mice ° p < 0.05, °° p < 0.01 and °°° p < 0.001 compared to saline control mice * p < 0.05, ** p < 0.01 and *** p < 0.001

In the early pathogenesis of DEN induced HCC, the mRNA expression of Epcam and Afp was continuously upregulated in all DEN treated mice compared to saline control (Figure 2A, B(Fig. 2)), strengthening the evidence for these characteristics as good markers of carcinogenesis (Chan et al., 2014[5]; Gomaa et al., 2015[13]). While no time dependent, PHD_2_ haplodefiency- relate effect could be observed concerning Epcam mRNA expression (Figure 2A(Fig. 2)), Afp expression was significantly increased after 15 weeks in PHD_2_^+/-^ mice compared to WT livers. However, this increased expression was not maintained after 17 weeks of DEN induction (Figure 2B(Fig. 2)).

DEN treatment resulted in significantly increased Prom1 expression in PHD_2_^+/-^ livers, compared to saline controls from 10 weeks onwards (Figure 2D(Fig. 2)) which was not observed for CK19. No difference between PHD_2_^+/-^ and WT counterparts could be observed for CK19 and Prom1 expression at any time-point (Figure 2C, D(Fig. 2)). Interestingly CK19 and Prom1 mRNA showed a peak expression after 17 weeks of DEN compared to Saline control and all other time points of the same genotype (Figure 2C, D(Fig. 2)).

Like Afp and Epcam, MDR1 mRNA expression was increased in all groups that received DEN compared to saline control (Figure 2E(Fig. 2)). Furthermore, MDR1 expression was significantly increased after 15 and 17 weeks of DEN, compared to all earlier time points in PHD_2_^+/-^ mice and compared to WT counterparts, and differed significantly between 15 and 17 weeks of DEN in WT livers (Figure 2E(Fig. 2)). 

**Figure 3:**
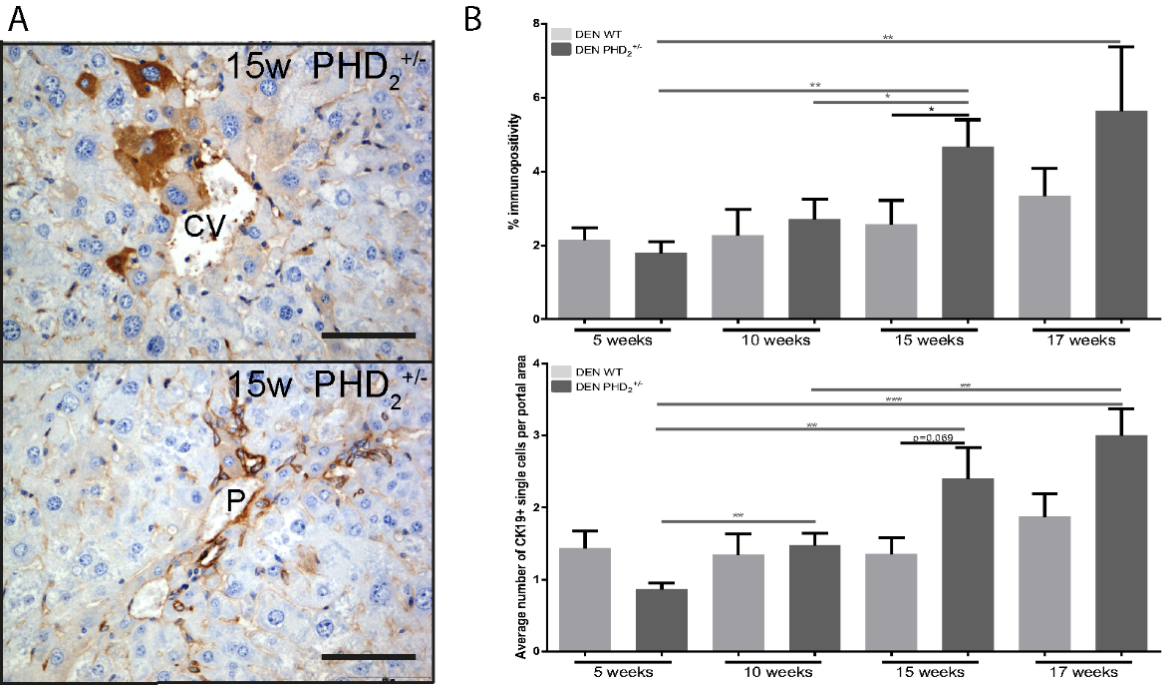
Cytokeratin 19 immunohistochemistry A. percent immunopositivity and average number of CK19 positive cells per portal area in PHD2+/- and WT counterparts at different time points in hepatocarcinogenesis B. CK19 immunopositive hepatocytes around the central vein after 15 week of DEN. CV: Central vein, P: Portal vein, Scale bars: 1000 µm, * p < 0.05, ** p < 0.01 and *** p < 0.001

Comparison of CK19 immunopositivity between PHD_2_^+/-^ and WT mice at different time points in hepatocarcinogenesis showed an increased number of central vein concentrated CK19+ single cells after 15 and 17 weeks of DEN (Figure 3B(Fig. 3)) and a tendency towards increased CK19 expression in PHD_2_^+/-^ mice at week 15 and 17 compared to earlier time points and compared to WT counterparts (Figure 3B(Fig. 3)). 

### Induction of the hypoxic adaptive response coincides with increased expression of Notch3 mRNA in early hepatocarcinogenesis

The Notch pathway plays a pivotal role in the cell-fate determination of LPCs and could also play a role in the increased expression of LPC characteristics observed after PHD inhibition. We therefore investigated the mRNA expression of Notch markers in early DEN- induced hepatocarcinogenesis in PHD_2_^+/- ^and WT counterparts.

We performed qPCR analysis of Notch receptors Notch1, 2 and 3, Notch ligand Jagged 1 (Jag1) as well as the main Notch effector gene Hairy enhancer of split 1 (Hes1). 

**Figure 4:**
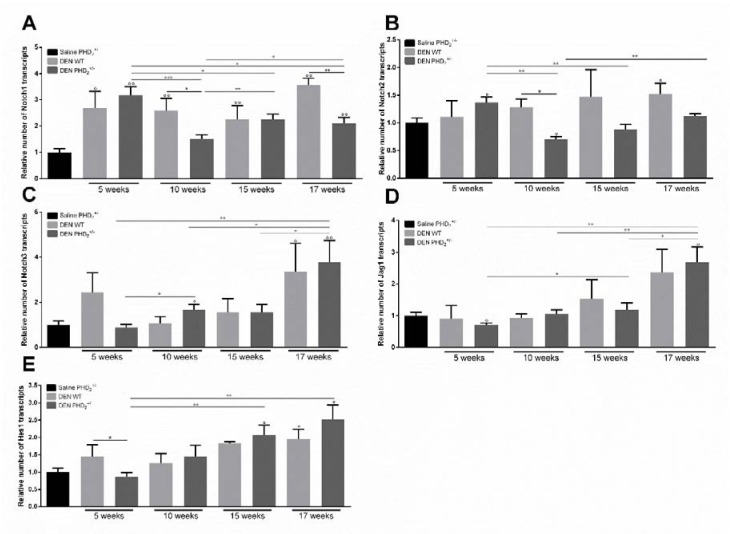
mRNA expression of Notch receptors and Notch target genes A. Notch1, B. Notch2, C. Notch3, D. jagged 1 (Jag1) and E. hairy transcriptor of split 1 (HES1) and in PHD_2_^+/-^ and WT mice, euthanised at different time points in hepatocarcinogenesis. °: p < 0.05, °°: p < 0.01 and °°°:p < 0.001 compared to saline control mice *:p < 0.05, **:p < 0.01 and ***:p < 0.001

DEN treatment did not induce consistent effects on Notch1 and Notch2 mRNA expression (Figure 4A, B(Fig. 4)). Expression of Notch3, Hes1 and Jag1 mRNA was significantly upregulated compared to saline control after 17 weeks of DEN in both WT and PHD_2_^+/-^ livers which coincides with increased expression of markers for hypoxia and HIF stabilisation (Figure 1(Fig. 1), 4C, D, E(Fig. 4)). However, no difference could be observed between PHD_2_^+/-^ and WT mice. After 17 weeks mRNA expression of Notch3 and Jag1 was also significantly upregulated in PHD_2_^+/-^ livers compared to same genotype livers at earlier time points (Figure 4C, D(Fig. 4)).

## Discussion

We have previously shown that in the DEN mouse model for hepatocarcinogenesis, PHD inhibition results in a mixed HCC-CC phenotype, high in LPC characteristics, which has been associated with a worse prognosis (Heindryckx et al., 2012[16]; Bogaerts et al., 2015[1]). In this study we aimed to investigate the effect of continuous PHD inhibition in early stages of hepatocarcinogenesis. We therefore used PHD_2_^+/-^ mice that were euthanised at different time points to unravel the dynamics of PHD_2_ haplodeficiency during early hepatocarcinogenesis, before nodule formation. 

We observed that PHD2 haplodeficiency did not result in altered liver morphology or onset of neoplastic transformation compared to WT controls. After 17 weeks of DEN, we observed a peak expression of HIF target genes, which was more pronounced in PHD_2_^+/- ^livers and coincided with the start of nodule formation as shown by histology. This allows the assumption that PHD haplodeficiency only affects gene expression of HIF target genes in the presence of hypoxia. 

We found that Afp and Epcam mRNA expression was continuously upregulated, in all DEN treated mice, at all observed time points. Indeed, Afp and Epcam have been shown to be expressed in hepatocytes during embryogenesis and in cirrhotic and cancerous livers (de Boer et al., 1999[7]; Ruck et al., 2000[32]; Yamashita et al., 2008[36]; Chan et al., 2014[5]; Gomaa et al., 2015[13]). The mRNA expression of CK19, Prom1 peaked after 17 weeks of DEN and coincided with increased expression of HIF target genes. Inherently to its microscopic structure, the liver can be divided in 3 zones, reflecting the level of oxygenation, with the hepatocytes around the central vein most prone to oxygen deprivation. The effects of PHD2 haplodeficiency will thus be most apparent in those cells. We did observe CK19+ hepatocytes around the central vein from 15 weeks onwards, further indicating that expression of CK19 could be related to increased HIF stabilisation. While it is unclear if CK19 and Prom1 expressing cells are progenitor cell derived (Govaere and Roskams, 2015[14]), or dedifferentiated hepatocytes (Mu et al., 2015[27]), recent studies have shown that increased CK19 and Prom1 expression in HCC is related to prognosis (Song et al., 2008[33]; Kim et al., 2011[20]; Govaere et al., 2015[14]) and recurrence (Song et al., 2008[33]; Zeng et al., 2012[41]).

Multi Drug resistance (MDR) proteins, which are inherently expressed by stem -and progenitor cells (Ros et al., 2003[31]) are drivers of therapy resistance and have been shown upregulated in hypoxic conditions (Comerford et al., 2002[6]; Krishnamurthy et al., 2004[21]), attributing to the observed poor prognosis for liver cancer with an increased progenitor and/or hypoxic signature. Interestingly, MDR1 mRNA expression was increased in all DEN treated mice, compared to saline control and in PHD_2_^+/-^ livers compared to WT counterparts from 15 weeks onwards, indicating that MDR1 could possibly be a marker for decreased PHD activity in early hepatocarcinogenesis. mRNA expression of MDR1 has, to our knowledge, not yet been mapped over time in animal models for hepatocarcinogenesis and its value as a potential marker for ongoing tumorigenesis and increased hypoxic signalling has not yet been explored.

Activation of the Notch signalling pathway has been shown to be involved in liver and other cancers, with contradictory proposed roles (Villanueva et al., 2012[35]; Capaccione and Pine, 2013[4]; Geisler et al., 2015[11]). Furthermore, while the distribution and prevalence of different Notch receptors have been reported in human healthy and diseased liver (Nijjar et al., 2001[28]) and in a murine model for experimental HCC (Huntzicker et al., 2015[18]), little is known about the expression of different Notch receptors in relation to phenotype and prognosis in experimental or human liver cancer. 

Inhibiting Notch 2 decreased HCC cell proliferation (Ortica et al., 2014[29]) and tumour burden (Huntzicker et al., 2015[18]), while Notch 1 and 2 overexpression in the liver resulted in spontaneous HCC development with biliary or LPC characteristics (Villanueva et al., 2012[35]; Dill et al., 2013[8]; Zender et al., 2013[40]). These observations are similar to the phenotype observed after PHD inhibition in HCC mice in previous studies (Heindryckx et al., 2012[16]; Bogaerts et al., 2015[1]) and as in human tumours recurring after TACE treatment (Zen et al., 2011[39]; Lai et al., 2015[22]). In this study we could not show altered Notch1 or 2 receptor mRNA expression during early hepatocarcinogenesis. Yet, we previously showed increased Notch2 mRNA expression, following PHD inhibition, associated with increased hepatocellular and cholangiocellular tumour burden at end stage DEN-induced carcinogenesis (Heindryckx et al., 2012[16]; Bogaerts et al., 2015[1]).

In PHD_2_^+/-^ and WT mice, Notch3 expression peaked after 17 weeks of DEN, like CK19 and Prom1 mRNA expression, indicating that expression of these markers coincides with nodule formation. This is in line with previous data, were Notch3 overexpressing HCC cells were shown to have increased aldehyde dehydrogenase activity (Zhang et al., 2015[42]), characteristic for LPCs (Dolle et al., 2012[9]) and inhibition was shown to overcome therapy resistance in HCC cells, increasing sorafenib toxicity (Giovannini et al., 2013[12]). 

In conclusion, we used PHD_2_^+/-^mice to evaluate the effect of increased HIF stabilisation during early hepatocarcinogenesis. However, increased HIF signalling was only observed during nodule formation, coinciding with increased mRNA expression of LPC characteristics and Notch3. We hypothesise that previously observed effects of increased HIF signalling on tumour phenotype manifest during tumour growth rather than development and are not preceded by an early LPC or Notch signature. Further elucidating possible mechanisms involved in this process could help to develop new therapeutic strategies to improve prognosis of patients with tumours growing in a fibrotic background, or receiving hypoxia- inducing therapies

## Acknowledgements

The authors would also like to thank Femke Wulgaert for her help with experiments as well as Anja Van den Bussche and Petra Van Wassenhove for their excellent technical assistance.

## Conflict of interest

The authors declare there is no conflict of interest.

## Supplementary Material

Supplementary figure

## Figures and Tables

**Table 1:**
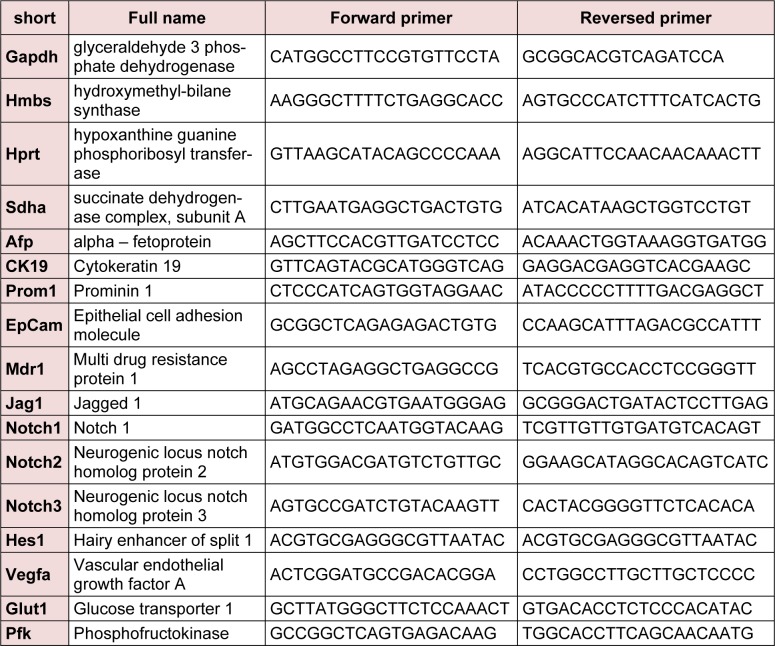
Primersets
