# Supplementary material for: Effect of prolyl hydroxylase domain 2 haplodeficiency on liver progenitor cell characteristics in early mouse hepatocarcinogenesis
Source: EXCLI J. 2016 Nov 11;15:687–98. doi: 10.17179/excli2016-607 (PMC5318796; doi:10.17179/excli2016-607)
Supplement: Supplementary figure [file EXCLI-15-687-s-001.pdf]

EXCLI Journal 2016;15:687-698 – ISSN 1611-2156
Received: August 26, 2016, accepted: October 13, 2016, published: November 11, 2016

Supplementary Figure

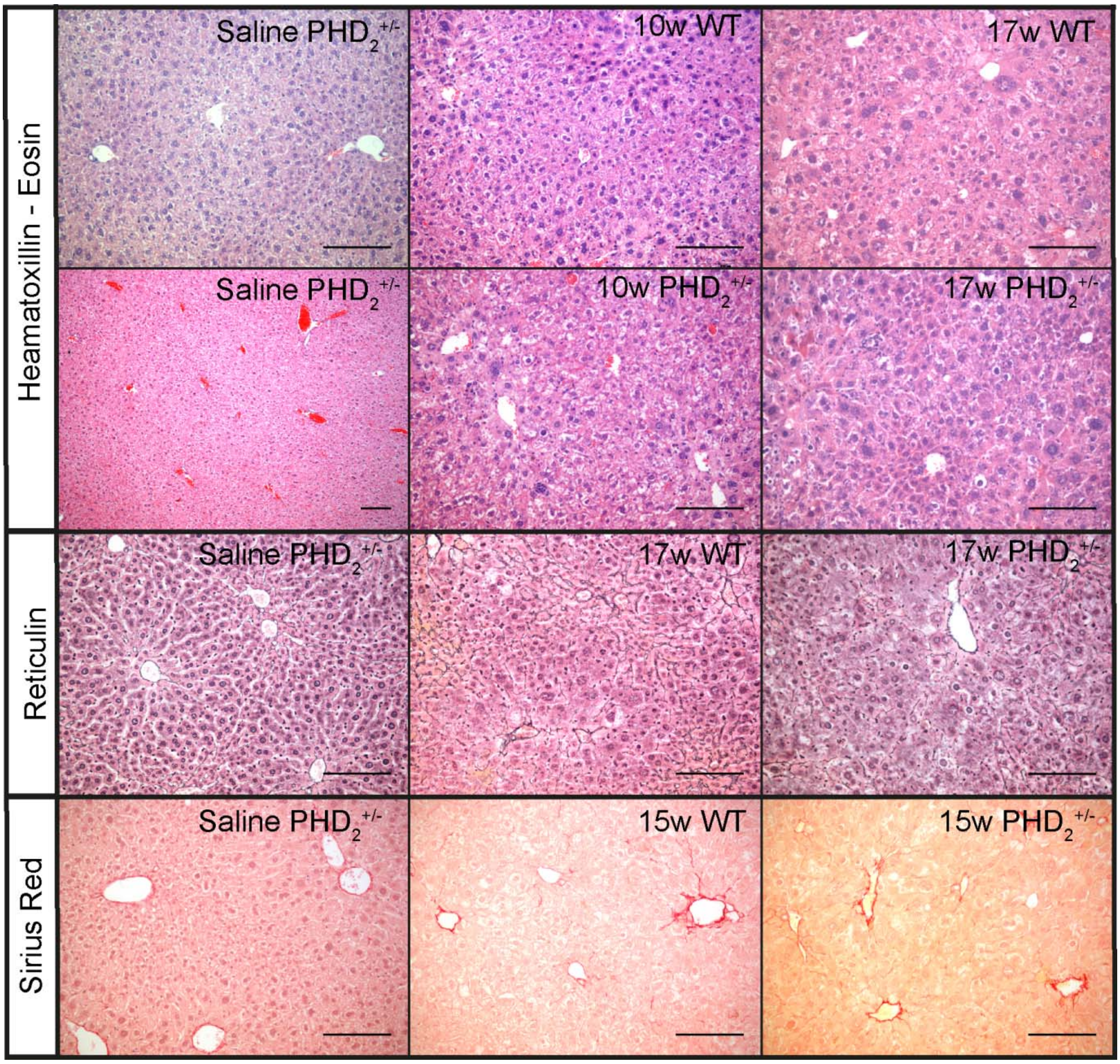

Figure S1: Representative images for Haematoxylin-Eosin, Reticulin and Sirius red stainings showing different groups at different time points. We observe neoplastic cells from 10 weeks onwards and loss of reticulin from 15 weeks onwards in PHD$_2^{+/-}$ and WT livers. Sirius red staining showed no presence of cholangiocytic lesions. Scale bars 2000 µm
